# Supplementary material for: Organogermanium THGP Induces Differentiation into M1 Macrophages and Suppresses the Proliferation of Melanoma Cells via Phagocytosis
Source: Int J Mol Sci. 2023 Jan 18;24(3):1885. doi: 10.3390/ijms24031885 (PMC9915250; doi:10.3390/ijms24031885)
Supplement: Supplementary file 1 [file ijms-24-01885-s001.zip › Supplementary Information.pdf]

Supplementary Figure Legends

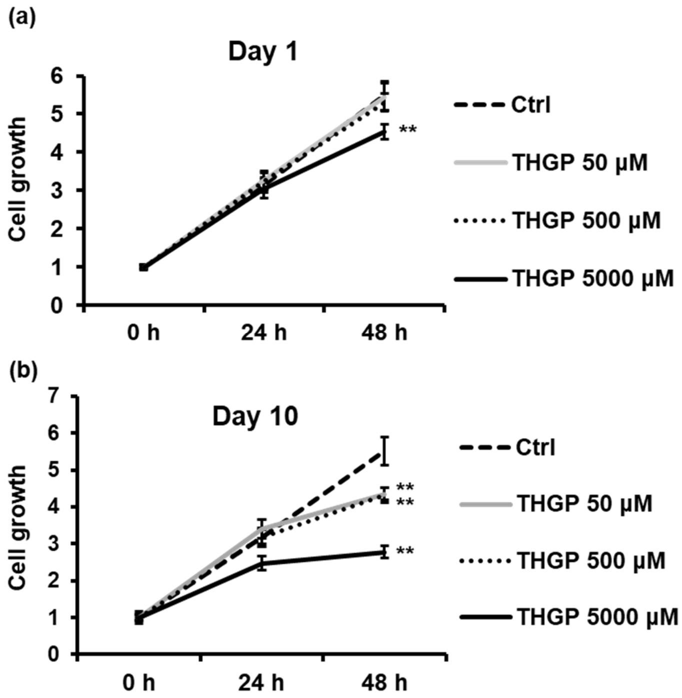

Figure S1. Growth of RAW 264.7 cells cultured with 50-5000 µM THGP.

The growth of RAW 264.7 cells cultured with 0-5000 µM THGP for 1 day (a) or 10 days (b) was analyzed using the MTS assay. ** $p < 0.01$.

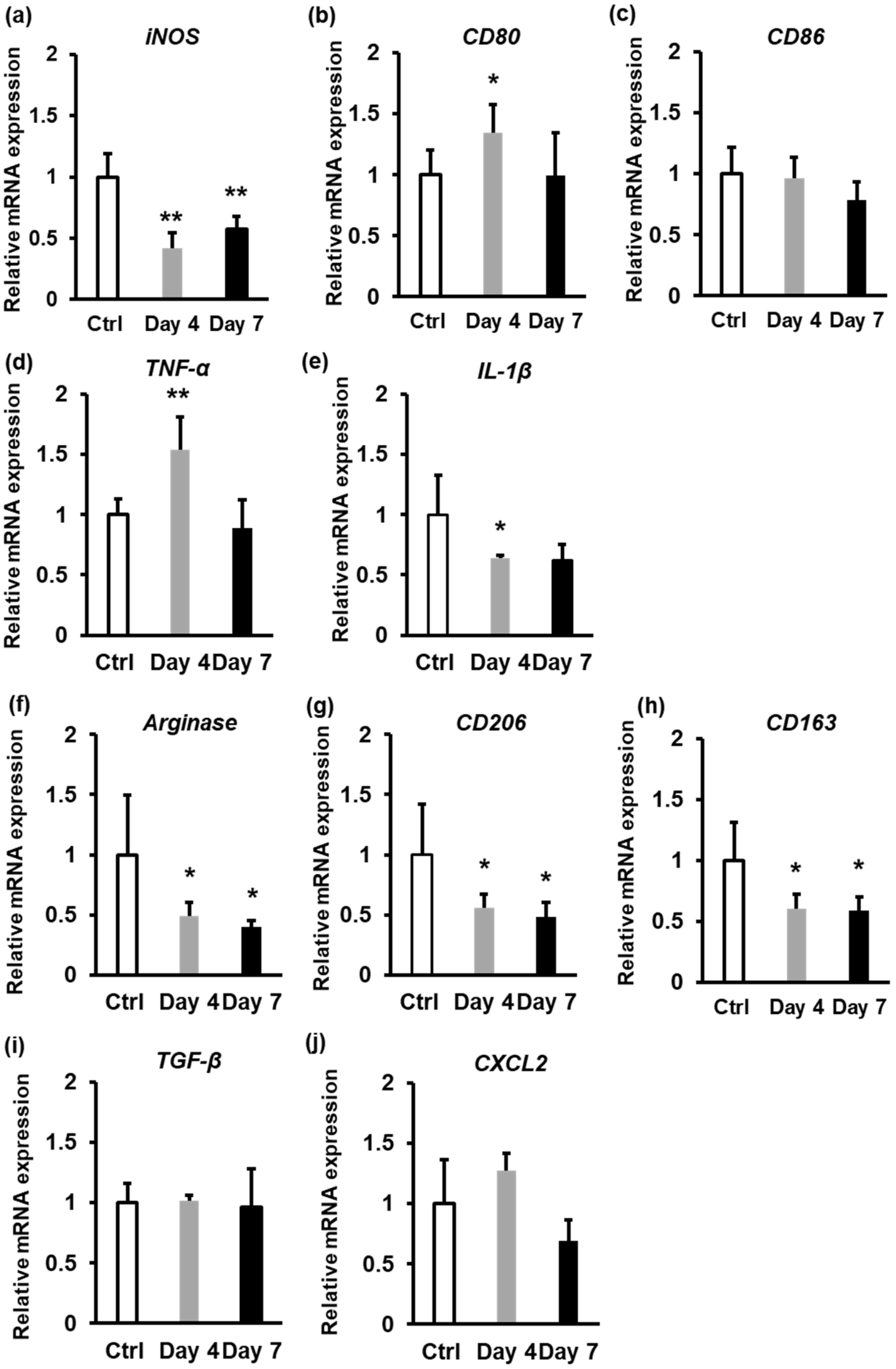

Figure S2. Expression of M1 or M2 markers in RAW 264.7 cells treated with THGP.

The expression of M1 markers (iNOS, CD80, CD86, TNF-α, and IL-1β) (a-e) or M2 markers (arginase, CD206, CD163, TGF-β, and CXCL2) (f-j) in RAW 264.7 cells

cultured with 500 µM THGP for 4 or 7 days was analyzed using RT–PCR. * $p < 0.05$, ** $p < 0.01$.

Figure S3. Expression of M1/M2 markers in macrophages from mice fed Ge-132

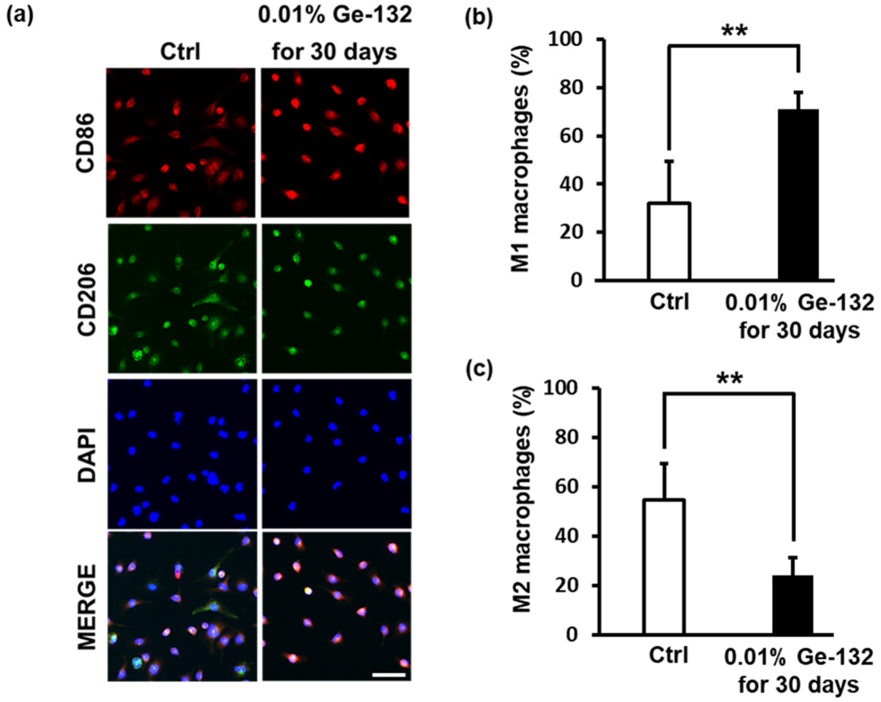

(a) Photographs showing immunofluorescence staining for CD86 (green) and CD206 (red) along with DAPI (blue) in mice fed a control diet or a 0.01% Ge-132 diet for 30 days. Scale bars: 20 µm. (b) M1 macrophages showed high expression of CD86 and low expression of CD206 in (a). (c) M2 macrophages with low expression of CD86 and high expression of CD206 in (a) n=6. ** $p < 0.01$ compared with the controls.

Table S1. Sequences of PCR primers.

Primers used for RT–PCR.

	**Forward (5'→3')**	**Reverse (5'→3')**
**iNOS**	CCCTTCAATGGTTGGTACATGG	ACATTGATCTCCGTGACAGCC
**CD80**	TGTCCAAGGCTCATTCTTCTC	TAACGGCAAGGCAGCAATA
**CD86**	AGCACGGACTTGAACAACCA	TGTAAATGGGCACGGCAGAT
**TNF-α**	TCAGCCTCTTCTCCTTCCTG	GGCTACAGGCTTGTCACTCG
**Arginase**	CTCCAAGCCAAAGTCCTTAGA	AGGAGCTGTCATTAGGGACATC
**CD163**	CTGGCGGGTGGTGAAAACA	CAGCCGTTACTGCACACTG
**CD206**	CTCTGTTCAGCTATTGGACGC	CGGAATTTCTGGGATTCAGCTTC
**CD47**	ATGCTTCTGGACTTGGCCTC	CCGACCAAAGCAAGGACGTA
**SIRPA**	CCACGGGGAAGGAACTGAAG	ACGTATTCTCCTGCGAAACTGTA
**IL-1β**	GCCTCGTGCTGTCGGACC	TGTCGTTGCTTGGTTCTCCTTG
**TGF-β**	ATTCCTGGCGTTACCTTGG	AGCCCTGTATTCCGTCTCCT
**CXCL2**	CCCAGACAGAAGTCATAGCCAC	GCCTTGCCTTTGTTCAGTATC
**RPS18**	TTCTGGCCAACGGTCTAGACAAC	CCAGTGGTCTTGGTGTGCTGA

Table S2. Microarray analysis.

Genes exhibiting more than a 4-fold increase or decrease in expression and a p value < 0.01 in the THGP-treated group compared to the control group are shown when analyzed using the microarray in Figure 3b. The data are shown as a Microsoft Excel file.

GO ID	Description	LogP	Enrichment	Z score	Gene no. in GO	No. of genes in GO and hitlist	No. of upregulated genes in GO and hitlist	No. of downregulated genes in GO and hitlist
TRR00951	Nfkb1	-6.5	3.6	6.5	246	22	6	16
TRR01204	Runx1t1	-4.2	30	9.3	4	3	2	1
TRR00652	Jun	-3.8	3.2	4.7	175	14	5	9
TRR01494	Trp53	-3.7	3.1	4.5	182	14	5	9
TRR00331	Etv4	-3.4	11	6	15	4	1	3
TRR01347	Sp1	-3.3	2.3	3.9	371	21	6	15
TRR00944	Nfe2l2	-3.3	4.9	4.7	57	7	4	3
TRR00327	Ets1	-2.9	4.3	4.3	65	7	4	3
TRR01190	Rela	-2.8	2.9	3.7	153	11	3	8
TRR00293	Egr1	-2.7	3.2	3.8	111	9	1	7

Table S3. TRRUST microarray analysis.

The table shows the results from the TRRUST analysis of genes showing a 4-fold increase or decrease in expression and p value < 0.01 in the THGP-treated group compared with the control group.

Movies. Movies showing cocultures of RAW 264.7 and B16 4A5 cells.

Two time-lapse videos captured from the coculture of RAW C (Movie S1) or RAW T (Movie S2) cells with B16 4A5 cells for 72 hours. The two other videos show magnified views of RAW C (Movie S3) or RAW T (Movie S4) cells phagocytosing B16 4A5 cells in Movie S1 or Movie S2, respectively.
